# Evaluation of a 3D-Printed Cleft Palate Obturator Using a Low-Dose Cone Beam Computed Tomography Acquisition Protocol: A Proof-of-Concept Study

**DOI:** 10.7759/cureus.57602

**Published:** 2024-04-04

**Authors:** Thomas Nelson, Santiago F Cobos, Vaibhav Gandhi, Bina Katechia, Sumit Yadav, Aditya Tadinada

**Affiliations:** 1 School of Dentistry, University of Connecticut Health, Farmington, USA; 2 Oral and Maxillofacial Radiology, University of Connecticut Health, Farmington, USA; 3 Orthodontics and Dentofacial Orthopaedics, Canadian Orthodontic Partners, Red Deer, CAN; 4 Pediatric Dentistry, University of Connecticut Health, Farmington, USA; 5 Growth and Development, University of Nebraska Medical Center, Lincoln, USA; 6 Oral and Maxillofacial Radiology, University of Connecticut, Farmington, USA

**Keywords:** 3d image reconstruction, cone-beam computed tomography, maxillary obturator, 3d digital models, cleft lip & palate

## Abstract

Cone beam computed tomography (CBCT) technology is increasingly utilized in the head and neck region and is valuable in treatment planning for cleft palate patients, potentially enabling the creation of 3D-printed obturators to assist with feeding and speech. This technical report investigates the feasibility of using data from a 360-degree CBCT scan to accurately produce a cleft palate obturator and assesses whether a lower-dose 180-degree CBCT scan can achieve a comparable result.

A simulated cleft palate was crafted on a dehydrated human skull, which was then scanned using both 360-degree and 180-degree CBCT scanning protocols. Two obturators were digitally designed based on the segmented images from each scan and subsequently 3D printed. Evaluation of the segmented images and 3D-printed obturators from both protocols demonstrated clear visualization of anatomical landmarks and identical scores across all parameters, suggesting that the 180-degree CBCT scan can produce an obturator of comparable quality to that of the 360-degree scan, with the added benefit of reduced radiation exposure.

## Introduction

Orofacial clefts, including cleft lip with or without cleft palate, are prevalent birth defects worldwide, occurring in approximately one in every 700 live births [1]. In the United States, cleft lip with or without cleft palate ranks as the second most common birth defect, with a prevalence of about one in 940 live births [2]. These conditions are associated with various complications, particularly during infancy and early childhood, such as feeding difficulties, swallowing challenges, speech impediments, ear diseases, hearing loss, and psychosocial issues [3]. Prior to surgical intervention, individuals with clefts often require an obturator to cover the palatal defect and facilitate proper feeding. Traditionally, obturator design involves taking a physical impression using conventional materials, but this approach can pose risks, including difficulty in removing the impression and potential respiratory obstructions, especially in neonates [4].

Various imaging modalities have been employed to enhance outcomes in cleft palate diagnosis and treatment, many of which are utilized during early developmental and active growth stages. These modalities include computed tomography (CT), cone beam computed tomography (CBCT), panoramic radiography, and occlusal radiography [5]. CBCT, a relatively recent diagnostic innovation developed in the late 1970s and adapted for dental applications in 1995, has gained increasing prominence in imaging the maxillofacial region, including for cleft lip and cleft palate conditions [6]. Both CT and CBCT provide three-dimensional (3D) visualization of bony structures and dental anatomy. Compared to conventional CT scans, CBCT offers several advantages, including lower cost, improved spatial resolution, better bone definition, and crucially, reduced radiation exposure [6]. This reduction in radiation exposure is particularly significant for young patients, who are more susceptible to the adverse effects of ionizing radiation and often require multiple imaging sessions in infancy and childhood [7].

A novel CBCT acquisition protocol involves a 180-degree rotation around the posterior area, from ear to ear, in contrast to the conventional 360-degree rotation. This modified protocol reduces radiation exposure by an average of 45% [8,9]. While the 180-degree acquisition scan exhibits a slight reduction in resolution due to fewer acquired basis images, it remains unclear whether this decrease in resolution would hinder its utility or render the images unsuitable for 3D printing applications, such as designing a cleft palate obturator.

## Technical report

Image acquisition

**Figure 1:**
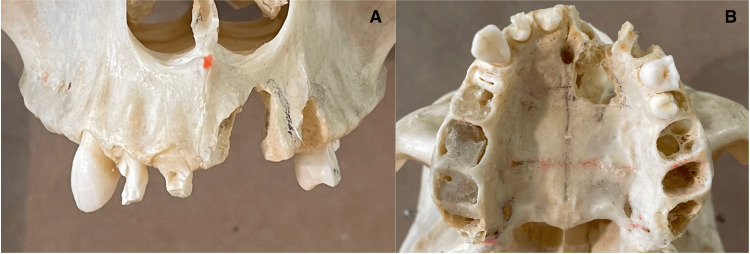
Simulated cleft palate on a human skull. A: Frontal view. B: Occlusal view.

One dry human skull was acquired from the Anatomy Department at UConn Health, and a high-speed dental handpiece was used to carve a simulated cleft palate into it (Figure 1).

The skull was positioned in a CBCT machine, Accuitomo 170 (J. Morita Corp., Kyoto, Japan), and was imaged with standard exposure parameters (90 kVp, 10 mA) with an 80 mm x 80 mm focused field of view. The acquisition time for the 360-degree scan was 19.8 seconds.

As a next step, without changing the position of the skull or the experimental setup, the low-dose scan with the 180-degree acquisition protocol was done. The exposure parameters with this protocol were 50 kVp and 2 mA with an acquisition time of nine seconds.

**Figure 2:**
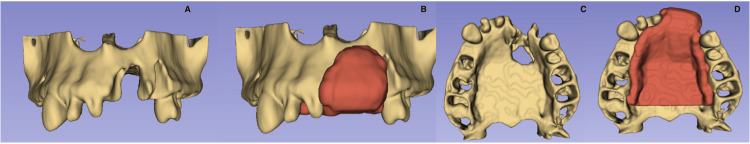
Segmented images of the skull derived from 360-degree protocol CBCT data with a designed obturator (red) fit to the segmented image. A: Frontal view without obturator. B: Frontal view with obturator. C: Occlusal view without obturator. D: Occlusal view with obturator. CBCT: cone beam computed tomography.

The 3D image volumes were reconstructed using Invivo CBCT reconstruction software (Anatomage, Santa Clara, CA). Then, the images were segmented using 3D Slicer software. Based on the 360-degree image data, an obturator was designed to cover the palatal defect communicating with the nasal cavity, the alveolar bone defect, and the remainder of the intact hard palate. Using the same external outline, an additional obturator was designed using the data from the 180-degree scan (Figure 2).

Printing

**Figure 3:**
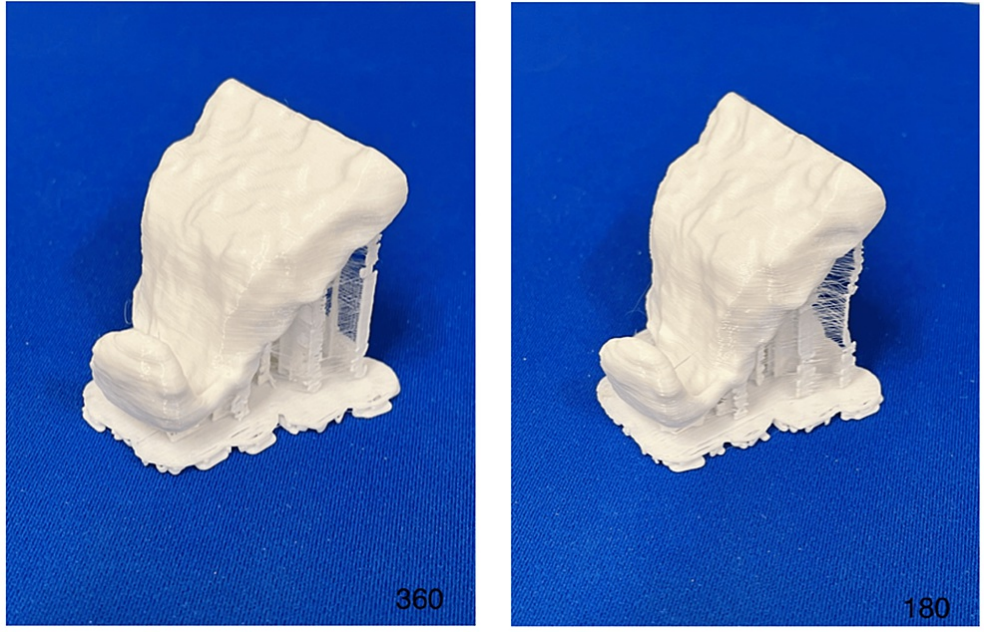
3D-printed obturators before support material removal designed from both the 360-degree CBCT protocol (left) and the 180-degree protocol (right). CBCT: cone beam computed tomography.

The files for both obturators were converted to STL file format. The STL data were used to print the designed obturators with a retail-grade MakerBot 3D printer (New York, NY) that uses fused filament fabrication. Standard retail-grade plastic printing material was used. The support material was removed, and each obturator was evaluated in the skull (Figure 3).

Results

**Figure 4:**
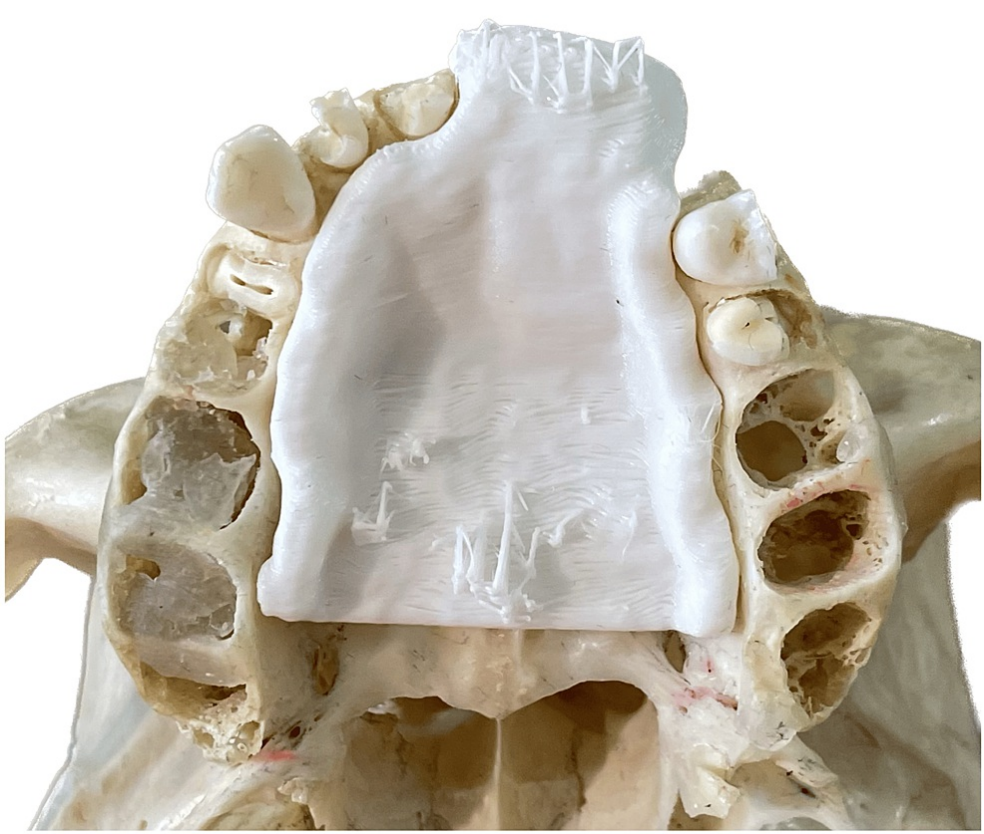
The 3D-printed obturator, designed from 360-degree protocol CBCT data, placed on the skull. CBCT: cone beam computed tomography.

Segmented images and the resultant obturator for each scan were evaluated with several scoring parameters. Scoring parameters for the segmented images included the ability to demarcate anatomical borders for segmentation, the presence of artifacts affecting anatomical depiction in either of the two scans, and the identification of various anatomical landmarks, including the maxilla, the nasopalatine canal, the extent of the hard palate, the buccal cortical plate, and the palatal cortical plate (Figure 4).

Scoring parameters for the 3D-printed obturators included the presence of obvious geometric distortions of the 3D-printed obturator in either of the two scans, ease of insertion of the obturator for each scan, and ease of removal of the obturator created from each scan.

In both images, anatomical borders were well-demarcated, no artifacts were detected, and all anatomical landmarks were clearly identifiable. Neither obturator exhibited any obvious geometric distortions, and both obturators could be easily inserted and removed. Differences between the two obturators were insignificant.

## Discussion

This study indicates that data obtained from a 360-degree CBCT scan are adequate for 3D printing a cleft palate obturator. Furthermore, the data from the lower-dose 180-degree CBCT scan showed no significant difference in quality for segmentation or design of the 3D-printed obturator.

However, designing an obturator for a live patient also requires consideration of soft tissue. Therefore, it is crucial to investigate whether the accuracy of an obturator remains acceptable when designed based on scans that include both hard and soft tissue. This is important because CBCT scans typically exhibit less soft tissue contrast than conventional CT scans [10]. Additionally, in practical applications, a softer printed material should be used to fabricate the obturator instead of the hard plastic used in this study from retail-grade 3D printers.

While a CBCT scan for a cleft palate patient can be recommended at various developmental stages, it can also be ordered in the early stages of life to aid in diagnosis [6]. If a CBCT scan is ordered for diagnostic purposes, the data from that scan could be directly used to print a highly accurate obturator, following a protocol similar to the one described in this study, without the need for additional procedures for the patient, such as physical impressions or intraoral scanning.

To further validate the feasibility of this method in clinical practice, a similar study incorporating the design of an obturator around soft tissue and using a material more suitable for human patients would be beneficial. Such a study would demonstrate that 180-degree acquisitions could replace 360-degree acquisitions for creating cleft palate obturators and other CBCT healthcare applications.

## Conclusions

In conclusion, this proof-of-concept study shows that a CBCT scan using a conventional 360-degree acquisition protocol can supply data for producing an obturator for cleft lip and palate patients. Furthermore, a comparable obturator can be created using a lower-dose 180-degree acquisition protocol.
